# Water clusters and density fluctuations in liquid water based on extended hierarchical clustering methods

**DOI:** 10.1038/s41598-022-11947-6

**Published:** 2022-05-16

**Authors:** Yitian Gao, Hongwei Fang, Ke Ni, Yixuan Feng

**Affiliations:** State Key Laboratory of Hydro-science and Engineering, Department of Hydraulic Engineering, Tsinghua University, Beijing, 100084 China

**Keywords:** Chemical physics, Thermodynamics, Chemical physics, Molecular dynamics

## Abstract

The microscopic structures of liquid water at ambient temperatures remain a hot debate, which relates with structural and density fluctuations in the hydrogen bond network. Here, we use molecular dynamics simulations of liquid water to study the properties of three-dimensional cage-like water clusters, which we investigate using extended graph-based hierarchical clustering methods. The water clusters can cover over 95% of hydrogen bond network, among which some clusters maximally encompass thousands of molecules extending beyond 3.0 nm. The clusters imply fractal behaviors forming percolating networks and the morphologies of small and large clusters show different scaling rules. The local favored clusters and the preferred connections between adjacent clusters correspond to lower energy and conformational entropy depending on cluster topologies. Temperature can destroy large clusters into small ones. We show further that the interior of clusters favors high-density patches. The water molecules in the small clusters, inside which are the void regarded as hydrophobic objects, have a preference for being more tetrahedral. Our results highlight the properties and changes of water clusters as the fundamental building blocks of hydrogen bond networks. In addition, the water clusters can elucidate structural and density fluctuations on different length scales in liquid water.

## Introduction

Water is the most ubiquitous liquid involved in multifarious physical, chemical, and biological processes on earth^1,2^. Different from simple liquids, liquid water shows numerous anomalous thermodynamic and kinetic behaviors, which are of significant importance to our planet and living systems^3,4^. At macroscopic temporal or spatial scale, liquid water can be regarded as a homogeneous substance. However, at microscopic scale, frequent spatial–temporal fluctuations emerge in the hydrogen bond network with local tetrahedral symmetry, elucidating the main reasons for anomalous properties of water^5^.

Water anomalies are strongly associated with locally favored structures corresponding to particular long-lived molecular arrangements with local minima of free energy^6,7^. Based on hydrogen-bonded networks, liquid water is a mixture of two different types of molecular arrangements: tetrahedral-like and hydrogen-bond distorted structures related with low- and high-density patches^8–12^, respectively. The presence of density fluctuations in hydrogen bond networks in supercooled or liquid water has been widely investigated according to experiments^13–18^ and molecular dynamics^12,19–22^. The model of two-state water, which comes from the competition between a high-density liquid (HDL) of distorted hydrogen bond patches and a low-density liquid (LDL) of ordered locally tetrahedral patches, can reproduce the anomalous behavior of thermodynamic properties of water^6,7,22^. Density heterogeneity occurs on a length scale of 10–15 Å at ambient temperature^14,15^. The presence of an isosbestic point at approximately 3450 cm^−1^ of the O–H stretching mode proves the coexistence of two types of local structures in liquid water by Raman spectroscopy^13^. In addition, X-ray small-angle scattering^14^, X-ray absorption^17^ and emission spectroscopy^18^ can provide evidence for inhomogeneous structures of liquid water. Based on molecular dynamics simulations, many models have been proposed to describe heterogeneous hydrogen bond networks to explain physical anomalous properties of liquid water, such as flickering models^23^ and percolation models^19,24^. Many structural descriptors have been proposed to quantify local favored structures in liquid water under various conditions, such as local structure index (LSI)^25^, the distance of five nearest neighbors (*d*_5_)^26^, and the sphericity of the Voronoi cell^27^.

Liquid water is a dynamical mixture of tetrahedral-like and “ring-and-chain”-like structures with a slight bias toward the former^19,28^, forming three-dimensional hydrogen bond network continually undergoing topological reformation^3^. Water clusters, an assembly of weakly-bound water molecules, may be formed in hydrogen-bonded networks by locally favored structures with local energy minima^29,30^. However, the microscopic structures of liquid water have remained a hot debate despite a lot of intensive research^15^. Graph theory is widely used to define topological structures as the building blocks of hydrogen-bonded networks^31–37^. Many investigations have introduced the definitions of rings^33^, fragments^34^ and clusters^35^. The rings, the cyclic paths along hydrogen bonds, show a broad distribution, among which 6-membered rings are most favored at ambient temperatures^31–33,38^. Fragments, three-dimensional cage-like building blocks constituted by rings, can elucidate the heterogeneity of hydrogen bond structures and rearrangements^34^. Topologically, water clusters also comprise small cyclic water clusters such as tetramers, pentamers and hexamers^39,40^. The topology of water clusters strongly affects the structure and chemistry of clusters^41,42^. The composite patterns of how flickering water clusters form bulk water remain a mystery despite several explorations^43–46^. A hierarchical clustering method^47^ is proposed to search out hierarchical water clusters in the hydrogen bond network. Hydrogen bonds, rings and fragments are defined as 1st-, 2nd-, and 3rd-level structures, respectively. Water clusters can be successively attained by using Louvain algorithm based on the network of fragments.

The local topological structures are closely related with density heterogeneity in liquid water at microscopic scale^48^. Many special structural patterns have been proposed to describe water structures and explain many anomalous properties^44–46,48–52^. Based on hydrogen bonds, fluctuating water clusters containing 280 water molecules with local icosahedral symmetry were proposed by Chaplin^46^. The networks contain a mixture of hexamer and pentamer substructures and convert between lower- and higher-density forms without breaking hydrogen bonds. A pair of liquid structures, molecular chains as high-density liquid structures and fused dodecahedra as tetrahedral fluctuations, are defined to reproduce physical properties^49^. In addition, a mixture of two types of helical clusters are also regarded as building blocks^44^. The empty spaces with hydrogen bond networks are strongly related with the nature of structural heterogeneities in liquid water^48–52^. Density fluctuations form a diverse morphology of voids, which can allow for the identification of low- and high-density patches of the liquid^48^.

In this paper, we aim to investigate the evolution of water clusters and the structural origin of density fluctuations using simulations with SPC/E. In our earlier work, we propose a graph-based hierarchical clustering methods^47^ to identify three-dimensional cage-like water clusters jointed by small-sized rings. Here, we further improve the hierarchical clustering methods to define water clusters covering most of hydrogen bond networks. Using classical molecular dynamics simulations, we study on how the water clusters changes at ambient temperatures. The distributions, morphologies, energetic and topological properties of water clusters are analyzed in detail. The characteristic structures of water clusters are further regarded as fundamental building blocks of hydrogen-bonded networks. By examining local number density and structural descriptor, the density fluctuations in the vicinity of water clusters are detected, proving that the water clusters can examine density fluctuations in the hydrogen bond networks, especially for small clusters.

## Results

### Molecular dynamics simulations and hierarchical clustering method

Molecular dynamics simulations are performed using periodic boundaries with 181,914 water molecules in a cubic box interacting through SPC/E water potential^53^. All of the simulations were run at temperatures of 278 K, 298 K and 318 K and a pressure of 1.0 atm with a Nose–Hoover thermostat and barostat. The time step was 1.0 fs. The systems were equilibrated for 2.5 ns in an NPT ensemble before outputting.

The hierarchical clustering methods^47^ respectively regard hydrogen bonds, rings, fragments as first-, second- and third-level sub-structures in the hydrogen bond network. The ring size is limited to 8 and the fragments analyzed contain 3, 4, and 5 rings. The graph community is a structure in which the nodes have a higher density of connections within groups than between them. Using Louvain algorithm^54,55^, the best graph communities are defined as water clusters, symbolizing the regions where the rings or specific structures are concentrated.

The alpha shape^56^ can describe the shape for water cluster by exploring the polytope wrapped by outermost water molecules. The volume *V* and surface area *A* of water clusters are defined as the volume and surface area of the alpha shape of clusters.

### Cluster statistics, morphologies and energy

The definition of ring-based clusters can intuitively describe the regions of cage-like local structures. In the hydrogen bond networks, over 95% of water molecules and over 60% of global box are involved in the formation of water clusters. Hence, the ring-based clusters can be regarded as specific microscopic structures in liquid water. It is essential to study the properties of water clusters to elucidate how the cage-like structures influence the hydrogen bond networks, and even physical properties of liquid water.

**Figure 1:**
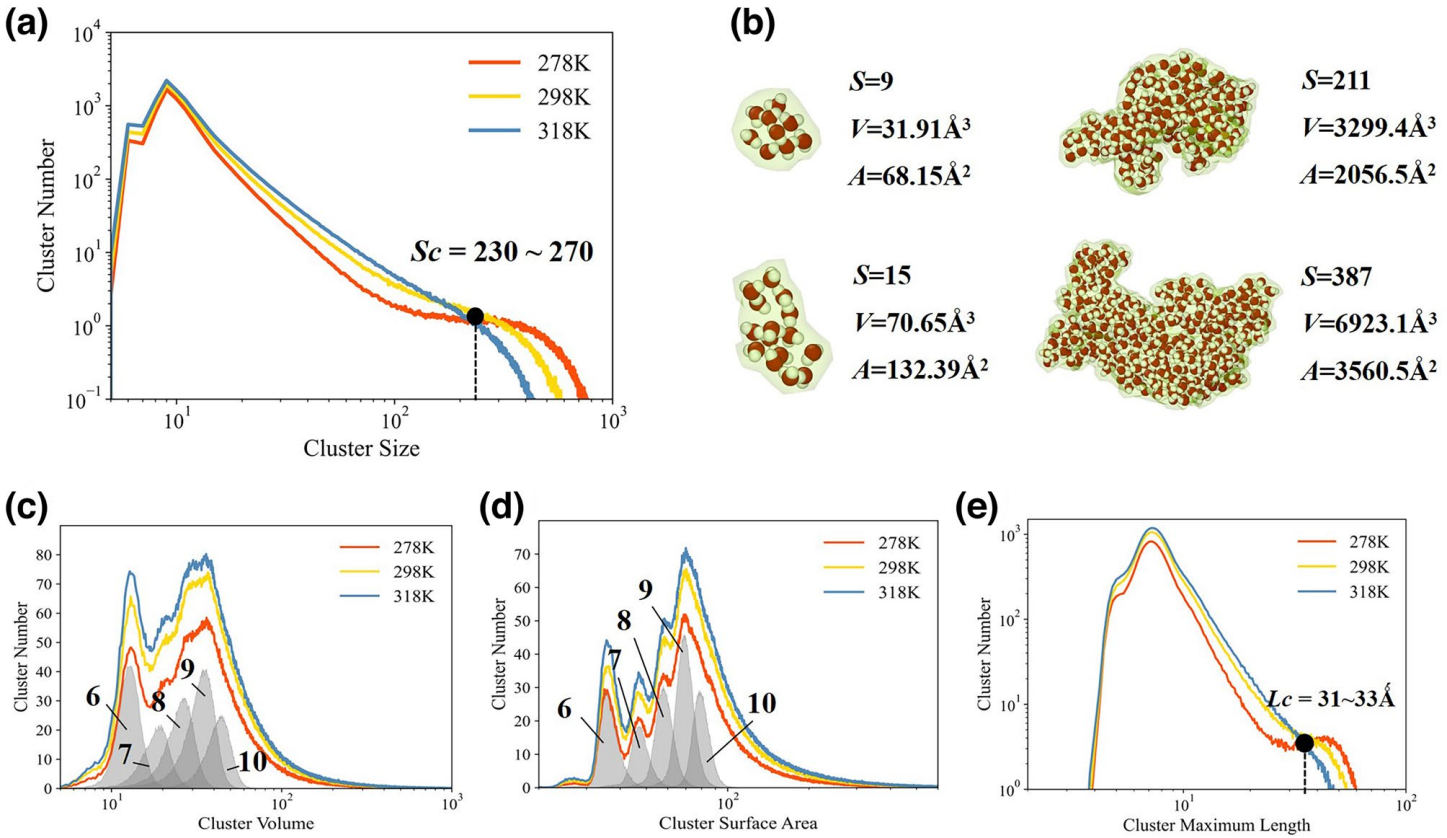
The cluster distributions and morphologies at various temperatures. (**a**) Water cluster distributions at 278 K, 298 K and 318 K. The black point denotes an isobestic point at *S*_*c*_ = 230–270. (**b**) Various water cluster morphologies at time step 0 at 278 K drawn by Ovito^58^. *S* denotes cluster size. *V* and *A* denote the volume and surface area of the alpha shape of clusters. (**c**) The volume distributions of water cluster at 278 K, 298 K and 318 K. The gray zones represent volume distributions of clusters with 6–10 molecules. (**d**) The surface area distributions of water cluster at 278 K, 298 K and 318 K. The gray zones also represent surface area distributions of clusters with 6–10 molecules. (**e**) The maximum length distributions of clusters at 278 K, 298 K and 318 K. The black point denotes an isosbestic point at *L*_*c*_ = 31–33°.

At ambient conditions, the hydrogen bond networks can form percolating networks which contain infinite hbond-based clusters spanning periodic cubic simulation box at least in one direction^57^. But in our definition, the ring-based networks do not have infinite clusters. The largest clusters only comprise more than 1% molecules in the box. Most of clusters are rather small, among which 6- and 9-molecule clusters are most favored in networks, as shown in Fig. 1a. Small clusters are mainly single-fragment clusters. Similar with hbond-based cluster distributions^57^, large ring-based clusters distribution also obeys a power law suggesting fractal behaviors. The distributions of *S* > 100 form a shoulder at low temperature.

Cluster morphologies, closely related with the voids inside clusters, allow for density fluctuations in the hydrogen bond network^48^. Some examples in Fig. 1b show various cluster morphologies. Small clusters tend to form spherical shapes and large clusters easily shape irregularly. As illustrated in Fig. 1c,d, the peaks of *V* and *A* for small clusters imply optimal cluster shapes implying subtle balance between hydrogen bonds enthalpies and structural distortions. But large clusters have broader distributions of *V* and *A* without distinct peaks suggesting complex morphologies. Meanwhile, as shown in Fig. 1e, maximum diameters of clusters *L*, the longest distance between two molecules in a cluster, can indicate that structural heterogeneity occurs on a length scale of more than 3.0 nm. 

**Figure 2:**
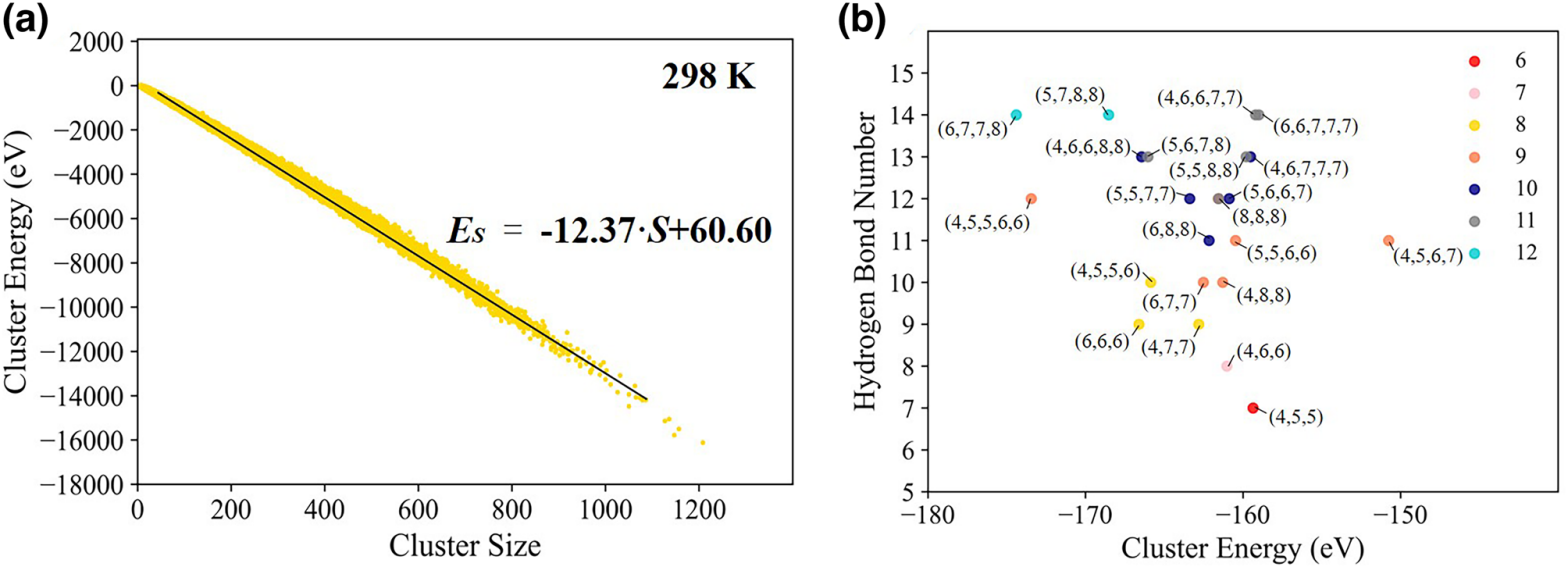
Scatter plots of cluster energy and cluster size and the relationship between cluster energy and cluster structures. (**a**) Scatter plots of cluster energy and cluster size at 298 K. The black line is the fit slopes by *E*_*S*_ = *a* × *S* + *b,* where *E*_*S*_ denotes cluster energy and *S* is cluster size. (**b**) The scatter of average cluster energy with certain cluster size and cluster structures. The colors represent cluster size. The labels of points represent ring components in a cluster.

Cluster energy plays an important role on the relationship between cluster structures and energy fluctuation in previous researches. The clusters have local energy minima based on quantum-chemical calculations and molecular dynamic simulations^59,60^. The energy of water clusters *E*_*S*_ is defined as the sum of interaction energy between molecules in the clusters. Under our definition of ring-based clusters in bulk water, the cluster energy *E*_*S*_ is almost directly proportional to cluster size *S* in form of *E*_*S*_ = *a* × *S* + *b*. The equation *E*_*S*_*/S* = *a* + *b/S* indicates that larger clusters may averagely have lower molecular energy *E*_*S*_*/S*, as shown in Fig. 2a. At the same time, cluster energy is mainly determined by hydrogen bonds number and cluster topologies related with the *quality* of hydrogen bonds, as illustrated in Fig. 2b. The formation of low-energy structures suggests local favored topologies, explaining that energy fluctuations in the hydrogen bond networks may be greatly connected with structural arrangements and dynamics.

Temperature can greatly change the properties of ring-based clusters. Cluster distributions have an isosbestic point *S*_*c*_ = 230–270 dividing cluster size with opposite temperature responses. With heating up, the cluster numbers decrease when *S* > *S*_*c*_ but increase when *S* < *S*_*c*_, suggesting that large clusters are destroyed into small ones and as a consequence average number of water clusters obviously increases. The isosbestic points are determined by box sizes shown in part IV of supplemental material. Then, it also indicates that the agglomeration of cage-like structures also tends to break up in the ring-based networks. Low temperature can cause the formation of lower-energy structures. In terms of *E*_*S*_ = *a* × *S* + *b,* at 278 K, 298 K and 318 K, the parameter a is equal to − 12.92 eV, − 12.37 eV and − 11.81 eV, respectively, and *b* is equal to 65.49 eV, 60.60 eV and 54.12 eV.

### Scaling behaviors of cluster distributions and morphologies

Fractals behaviors are the intrinsic nature of microscopic particles and networks to describe self-similarity. The infinite clusters at the percolating threshold of hydrogen bond networks belong to fractal object showing scaling behaviors of distributions. The morphologies of the cavities of Lennard–Jones fluid^61,62^ and the voids of hydrogen bond networks in liquid water^48–52^ also show obvious scaling behaviors suggesting unde rlying fractal structures.

The percolating threshold demonstrates the existence of percolating transition forming infinite hydrogen-bonded clusters, which closely relates with phase transition, especially liquid–liquid transition in supercooled water^52,57^. At ambient conditions, the ring-based networks do not reach percolating threshold forming infinite cage-like clusters. The ice can form infinite ring-based clusters because the networks are tessellated by tetrahedral structures. It implies that percolating transition of ring-based networks may cause the formation of infinite cluster at lower temperature than hydrogen bond networks. The infinite clusters can represent specific chain- or sheet-like structure of spanning hydrogen-bonded network at mesoscopic scale. Formation of giant cluster structures may affect the properties of liquid water which are sensitive to mesoscopic structures^57^. The ring-based clusters also showing scaling behaviors of distributions and large cluster distributions also obeys a approximate power law *n*_*S*_ ~ *S*^*−τ*^, where *n*_*S*_ denotes cluster number and *S* denotes molecular number in a cluster. The fractal dimensions *τ* are roughly equal to 2.44, similar to exponents *τ* = 2.20 in the case of hydrogen bond networks^57^.

**Figure 3:**
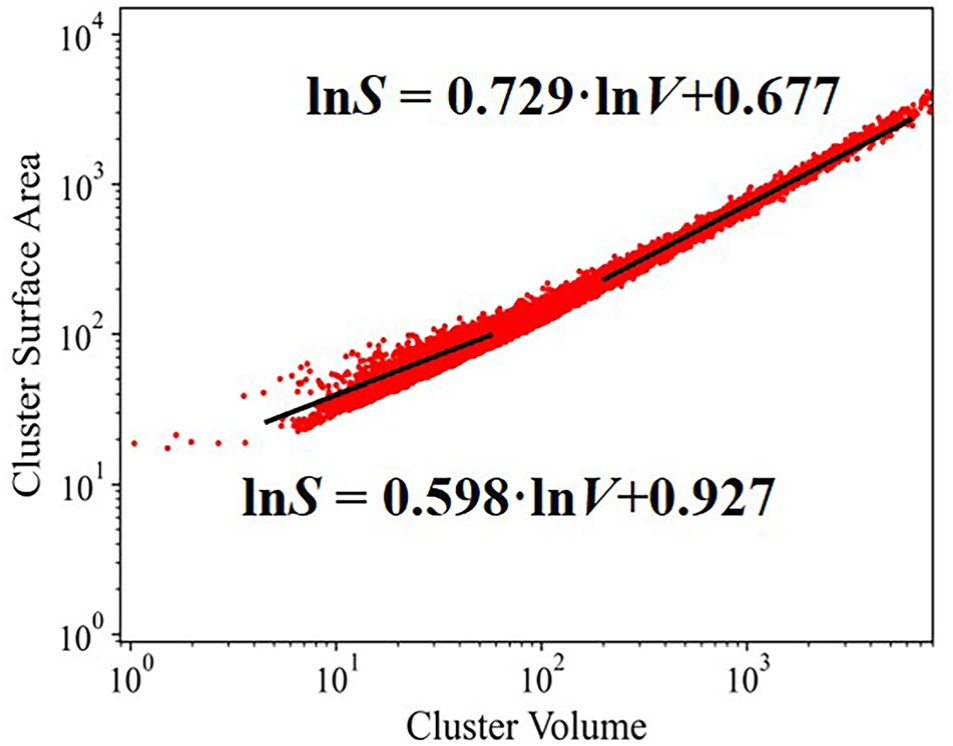
The log–log scatters of cluster volume and surface area. The black line is the fit slopes by *lnS* ~ *alnV* + *b.*

The complex morphologies of water clusters also show underlying fractal structures. Both cluster volume *V* and surface area *A* scale well with cluster radius *r* = *L*/2 illustrated in Fig. 3, giving *d*_*V*_ = 2.89 and *d*_*S*_ = 2.01. It indicates that the cluster shapes are self-similar. The surface-to-volume scaling of clusters also show scaling behaviors in form of a power law ln*A* ~ *a*ln*V* + *b*^63^. Similar with voids in hydrogen bond networks, small and large clusters show different scaling characteristics. For small clusters with *V* < 100 Å^3^, *a* = 0.598,* b* = 0.927; for large clusters with *V* > 100 Å^3^, *a* = 0.729,* b* = 0.677. The small clusters are non-fractal three-dimensional objects because the parameter *a* is near 2/3. Then, large clusters slightly display surface fractals because the parameter *a* lie between 2/3–1. The shape factor *b* depends on the shape, e.g. for spheres *b* = 1.57, for cubic cavities *b* = 1.79, for tetrahedra *b* = 1.97.

### The dynamics and transformations of water clusters

The thermal fluctuation cause frequent arrangement of hydrogen bond network and then the recombination of water clusters. Accordingly, the cluster lifetime is very transient and can averagely last for ~ 10 fs, compared with the hydrogen bond lifetime of approximately 11.79 ps, 7.99 ps and 5.84 ps at 278 K, 298 K and 318 K. Without any assumptions of lifetime^64^, average lifetime of clusters with *S* < 10 is approximately 4.0Δ*t*, where Δ*t* denotes the timestep Δ*t* = 10.0 fs. The average lifetime of clusters with *S* > 50 is only 0.39Δ*t*. Temperature also can shorten the lifetime of the clusters with *S* < 10, 4.0Δ*t* at 278 K, 3.7Δ*t* at 298 K and 3.4Δ*t* at 318 K.

**Figure 4:**
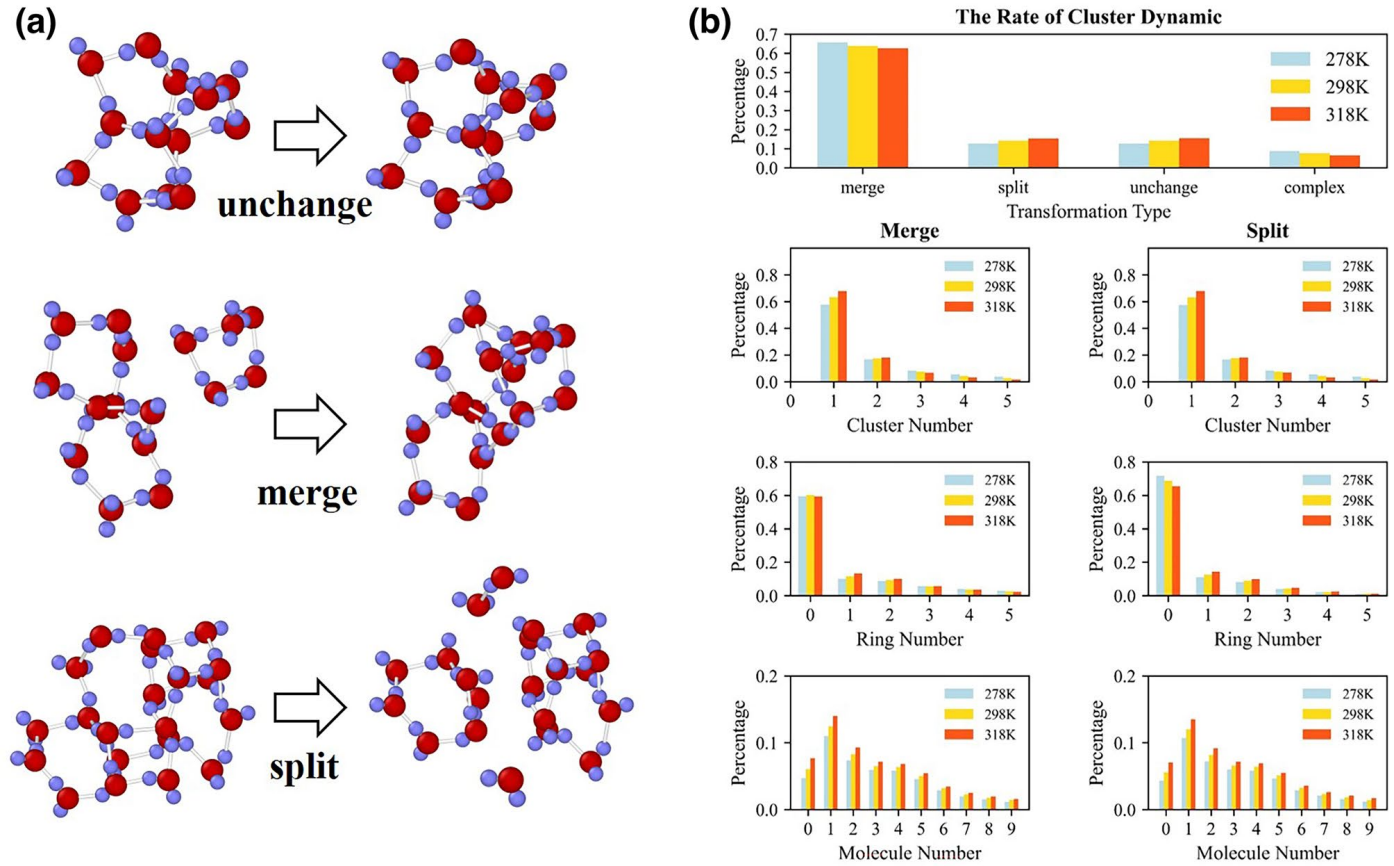
Example of cluster transformation and the distribution of cluster transformation at various temperatures. (**a**) Some example of cluster transformation involving unchanged, merged and split at 298 K drawn by Ovito^58^. (**b**) The rate of cluster transformation and the products or reactant distributions of cluster splitting and merging at 278 K, 298 K and 318 K.

Cluster transformations have four patterns: changeless, merge, split and complex fusion and fission, as illustrated in Fig. 4a. Assume that a cluster, a ring and a molecule that belongs to the debris of rings or clusters at timestep *t* are defined as *C*_*t*_, *R*_*t*_ and *m*_*t*_.

1. Changeless: the cluster remains changeless in neighboring configurations.
  1 $${1}{\text{C}}_{t}\Rightarrow {1}{\text{C}}_{t+1}$$
2. Merge: A cluster is constituted by at least one cluster or one ring at previous configurations.
  2 $${i}_{c}{C}_{t}+{i}_{R}{R}_{t}+{i}_{m}{m}_{t}\Rightarrow {1}{\text{C}}_{t+1}\left(\begin{array}{ccc}{i}_{c}\ge 1& or& \left.{i}_{R}\ge 1\right)\end{array}\right.$$
3. Split: A cluster is transformed into at least one cluster or one ring in the next configurations.
  3 $${1}{\text{C}}_{t}\Rightarrow {i}_{c}{C}_{t+1}+{i}_{R}{R}_{t+1}+{i}_{m}{m}_{t+1}\left(\begin{array}{ccc}{i}_{c}\ge 1& or& \left.{i}_{R}\ge 1\right)\end{array}\right.$$
4. Complex fusion and fission: None of the transformations belong to the above types.
  4 $${i}_{c}{C}_{t}+{i}_{R}{R}_{t}+{i}_{m}{m}_{t}\Rightarrow {{i}^{{\prime}}}_{c}{C}_{t+1}+{{i}^{{\prime}}}_{R}{R}_{t+1}+{{i}^{{\prime}}}_{m}{m}_{t+1}$$

In the 10-fs intervals, over 60% of clusters remain unchanged as shown in Fig. 4b. The rates of merging and splitting are comparatively equivalent about 12.0–16.0%. The reactants of cluster merging are mostly 1 cluster and 1–5 molecules. Only 30% merging contain isolated rings. The products of split are also 1 cluster and several molecules. High temperature can decrease the rate of unchange clusters and increase the rate of merging and splitting from 12.7% at 278 K to 15.4% at 318 K. The cluster transformations are very frequent and prefer to merge or split the debris of other clusters, instead of ring structures, suggesting randomness of cluster dynamics. The cumulative effects of clusters cause the change of local structures and then complete rearrangements of hydrogen bond networks.

### Structural heterogeneity of water clusters

In the hydrogen bonded networks, the water clusters represent ring-concentrated regions where different constitution of ring structures implies structural heterogeneity closely related with the arrangement of hydrogen bond networks.

**Figure 5:**
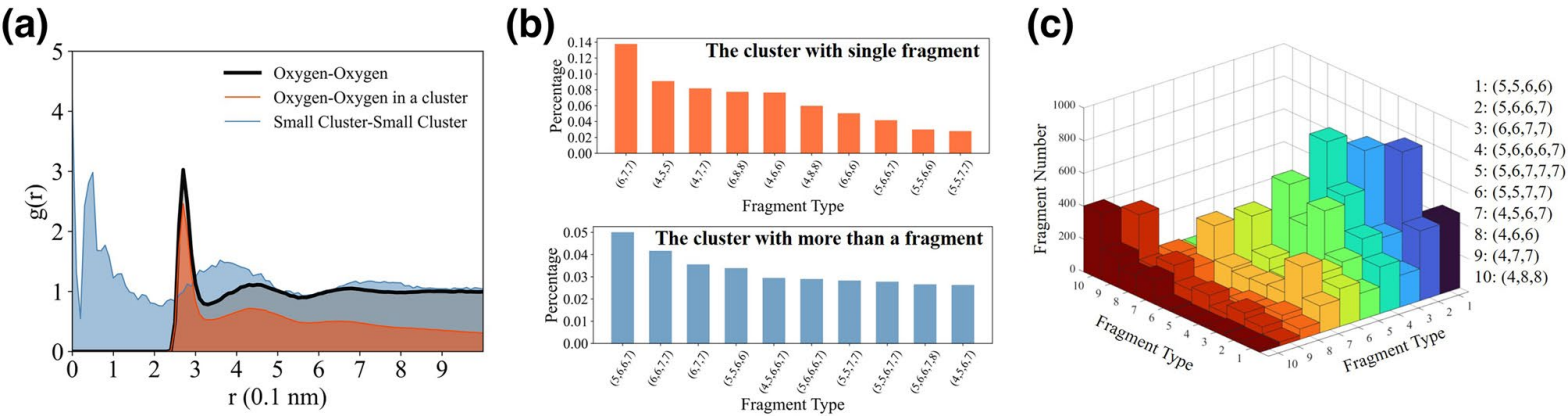
The oxygen–oxygen and cluster–cluster radial distance functions and topological properties of clusters. (**a**) The radial distance functions of oxygen–oxygen in global system, oxygen–oxygen in large clusters and small cluster-small cluster. The cluster RDF is calculated by the mass point of clusters with no more than 10 molecules. (**b**) The top 10 favored fragments in the clusters with a fragment and more than one fragment at 278 K. (**c**) The distributions of adjacent fragments in the clusters. Labels 1–10 represent most local favored fragments in the hydrogen bond network.

The spatial distributions of clusters suggest microscopic local structures between clusters. The RDFs (radial distribution functions) of oxygen–oxygen atoms in the clusters have several peaks at 2.7 Å, 4.3 Å and 6.5 Å similar with that of all oxygen atoms and gradually approach zero, as illustrated in Fig. 5a. For small clusters, the RDF peaks are located at 0.5 Å, 3.6 Å and 7.2 Å in Fig. 4d, respectively representing adjacent water clusters. It indicates that structural inhomogeneity around ring-concentrated regions reveal weak structuring up to ~ 1 nm.

The cluster structures have several favored topologies. Several fragments are locally favored because of large quantities and long lifetimes in part II of supplemental material. Several favored patterns of fragments involve (6,7,7), (6,8,8), (4,7,7), (4,6,6), (6,6,7,7), (5,6,6,7), (5,6,6,7,8) and so on. The more 5- and 6-membered rings in the clusters gain in popularity with local structures. Most clusters have only one fragment, 59.1% of which contain 3 rings. Over 86% clusters with more than one fragments have 4 and 5 rings. The 3-ring fragments are easily isolated. The 6-molecule clusters belong to (4,5,5) fragments. Most 9-molecule clusters were (6,7,7) and (4,8,8) fragments. The 4- or 5-ring fragments tend to aggregate into large clusters including (5,6,6,7) and (6,6,7,7), as shown in Fig. 5b.

Figure 5c shows the probability of different clusters adjacent to each other. It reveals that the fragments prefer to adjoin same type of fragments, for instance 3-ring fragments tend to combine 3-ring fragments. The top 5 favored connection modes between cluster with 2–6 fragments at 298 K are also charted in part III of supplemental material. Strong tendency of certain pairs of fragments being adjacent in the clusters accounts for lower conformational entropy of connected structures in liquid water.

The surface rings among adjacent clusters have significant effects on the compatibility among adjacent fragments. The rings connecting two fragments in a cluster are defined as body rings, and the others are defined as surface rings. The 55% of 4-membered rings and 45% of 6-membered rings bridge adjacent fragments. The surface rings, where most hydrogen bond arrangements occur, dominate at high temperature. When the clusters are formed, the defects are transformed from their interior to surface area. The dynamics of surface area can cause the growth or decay of ring-concentrated regions^34^.

### Water clusters and density fluctuations in the hydrogen bond network

The water clusters can represent ring-concentrated cage-like structures. We explore how the water clusters are related with density fluctuations and then prove that water clusters can be regarded as characteristic structures to describe inhomogeneous patches of hydrogen bond networks on different length scales.

First, local density (*ρ*_*local*_) near the center of clusters is a significant parameter to quantify density fluctuations around cluster structures. The local density (*ρ*_*local*_) around the center of a cluster is defined as the number of oxygen atoms inside a probe sphere with a radius of 4.6 Å^48^. The bulk number density at 298 K fluctuates at approximately 0.0334 molecule/Å^3^. The small and large water clusters respectively have *S* ≤ 10 and *S* ≥ 100. The average maximum cluster radius is approximately 3.85 Å and 19.40 Å.

**Figure 6:**
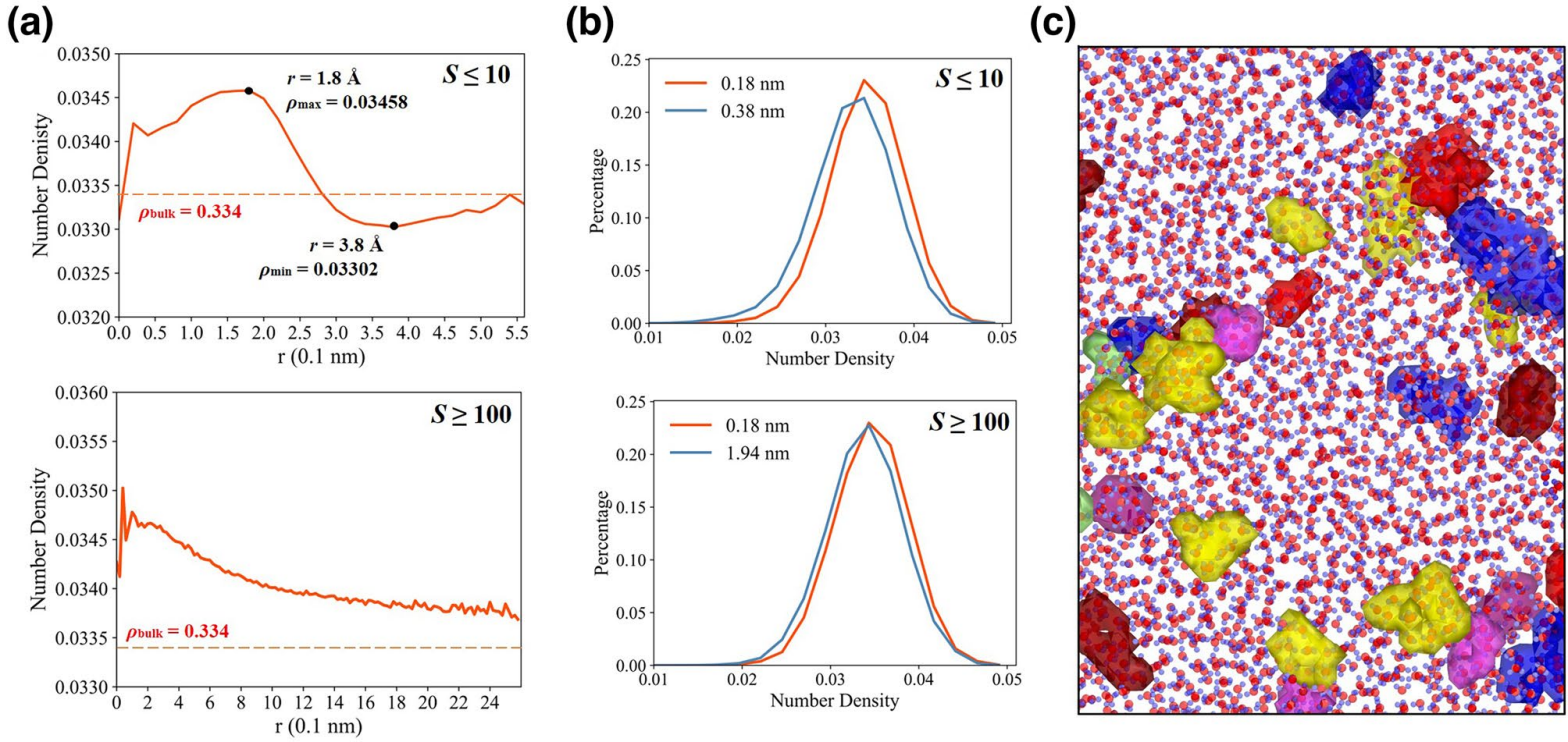
High- and low-density patches around the geometrical center of clusters at 278 K. (**a**) The number density around the geometrical center of clusters with *S* ≤ 10 and *S* ≥ 100, which denotes molecular number. The number density is calculated as the number of oxygen atoms inside a probe sphere located at certain oxygen atoms in the clusters with a radius of 4.6 Å. (**b**) The distributions of number density located at maximum density and minimum density. (**c**) Snapshots of several clusters selected in local region drawn by Ovito^58^.

As shown in Fig. 6a, high-density patches exist in the interior of clusters. For small clusters, the distribution of densities reaches the peak at 1.8 Å and the minima near clusters surface. High-density patches is ~ 3% larger than bulk density. The regions outside the interior of large clusters are still larger than bulk density. In Fig. 6b, for small and large clusters, 64.8% and 64.7% local densities are larger than the bulk density at density peak compared with 51.3% and 57.4% at minima, which explain the discrepency of density at different regions. Figure 6c shows an instantaneous snapshot of large cluster at 278 K, illustrating spatial inhomogeneities of different types of clusters.

The formations of structuring of water molecules have an implication on hydrophobic effect in liquid water^65^. The hydrophobic solvation may create an empty cavity in the hydrogen bond network and form stronger inter-hydrogen bonds^66^. To eludicate how water molecules behave inside the clusters, the local structure index (LSI)^25^ has been introduced to characterize local structures in liquid water^7,12,22,48^, measuring the extent to which the gap opens between water molecules in the first shell. LSI is defined as,

5 $$LSI=\frac{1}{n}\sum \limits_{i=1}^{n}{\left(\Delta \left(i\right)-\overline{\Delta }\right)}^{2}$$

$\Delta (i)={r}_{i+1}-{r}_{i}$ and $\overline{\Delta }$ is the average over all molecules in the first shell. The molecules are numbered in order of distance from the center molecule, ${r}_{1}<{r}_{2}<\cdots<{r}_{i}<3.7$ Å <* r*_*i* + 1_. Large LSI values represent tetrahedral water molecules, whereas small LSI values correspond to disordered first shells^21^.

**Figure 7:**
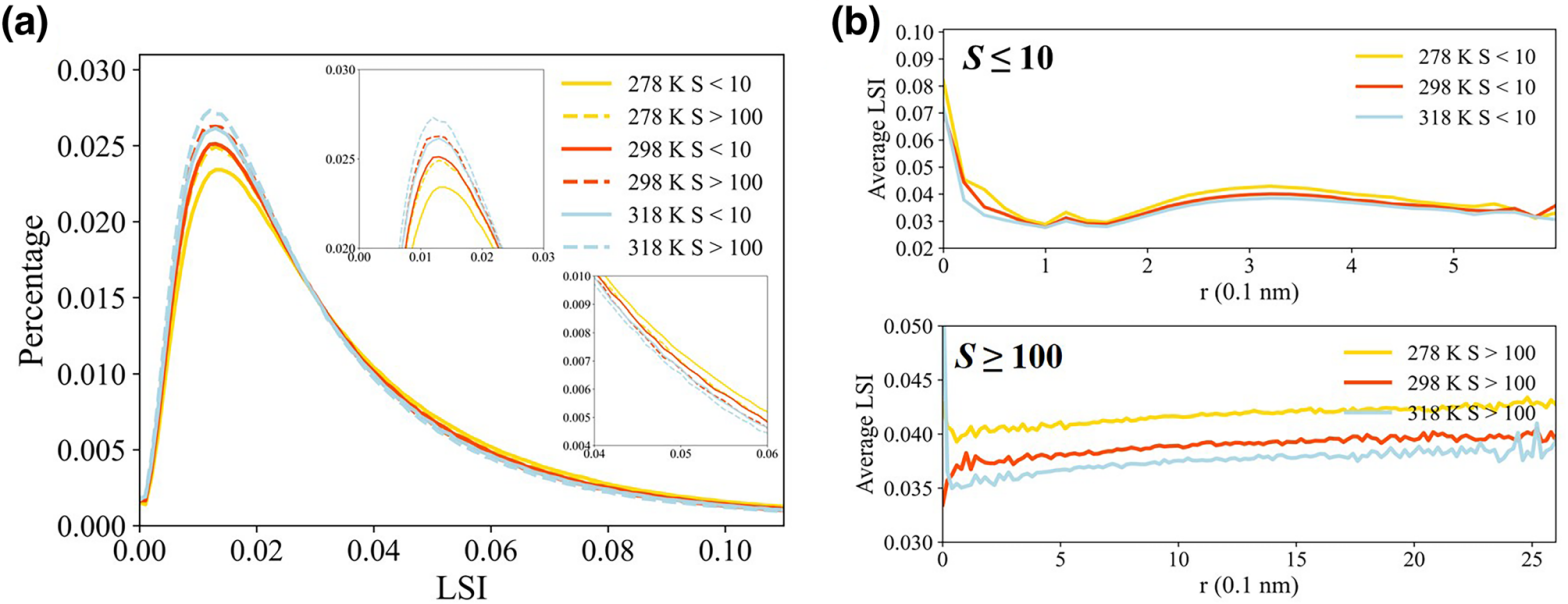
The distribution of LSI for waters in the vicinity of the clusters at various temperatures. (**a**) The distribution of the LSI of water molecules in the clusters at 278 K, 298 K and 318 K. The solid line denotes the clusters with *S* ≤ 10, and the dashed line denotes the clusters with *S* ≥ 100. (**b**) The average LSI around the geometrical center of clusters with *S* ≤ 10 and *S* ≥ 100.

In Fig. 7a, the tetrahedrality of water molecules decreases with increasing temperature. The isosbestic point at *LSI* ≈ 0.03 Å^2^ can divide two features of structures, representing the bimodality of LSI distributions having high-density- and low-density-like local structural environments^21^. The average tetrahedrality for water molecules in small clusters is slightly larger than that in large clusters. The voids inside small clusters, can be regarded as hydrophobic objects and hence the water molecules on small cluster surface have a preference for being more tetrahedral. The local structures of water molecules in the clusters show spatial nonuniformity and the tetrahedrality is negatively correlated with densities along the radius of clusters. The high-density patch in the clusters correspond to higher tetrahedrality, implying a more ordered first shell environment near non-polar hydrophobic solutes. It is also consistent with the results that long-range structural fluctuations of patches of four-coordinated molecules form from the liquid to supercooled water^67^.

There is compositional similarity between the water clusters and voids. The water molecules in the clusters can encircle the voids wrapped in the closed rings. The small clusters may form spherical voids; moreover, the large clusters may involve some void clusters. Both structures show similar properties of distribution and morphology. Similar with water molecules around the voids^48^, small clusters reveal similar correlations with density fluctuations. The deeper relationship between water clusters and voids needs to be explored further.

## Conclusions and discussions

The microscopic structures in liquid water remain a hot debate in recent researches. We search out water clusters in the hydrogen bond networks of liquid water using 100-thousand-particle molecular dynamic simulations by hierarchical clustering methods.

In the present paper, we show that there is underlying correlation between hydrogen bond networks and the properties of ring-based clusters within it. We demonstrate that the water clusters can intuitively identify density and structural fluctuations in the hydrogen bond networks, which leads to microscopic interpretations of waters anomalies. In particular, we show that the regions inside the clusters correspond to high-density patches with lower tetrahedrality, especially for small clusters. The small clusters have a preference for having more tetrahedral molecules. It implies a more ordered first shell environment near non-polar hydrophobic objects which is equivalent with the voids in small clusters. And we also show that some clusters are favored by hydrogen bond networks depending on hydrogen bond numbers and cluster topologies. The compatibility of adjacent clusters depends on conformation and size of surface rings bridging them. The structural preference for water clusters also explain the arrangement and dynamics of hydrogen bond networks.

The ring-based clusters in percolating hydrogen bond networks show scaling behaviors of cluster distributions and morphologies forming underlying fractal structures and self-similarity. We show that small clusters are non-fractal three-dimensional objects and large clusters slightly display surface fractals. And large clusters distribution obeys power law with fractal dimension equal to 2.44. It also implies that percolating transition of ring-based networks may cause the formation of infinite cluster at lower temperature than hydrogen bond networks. Also, temperatures largely influence cluster properties and dynamics in the hydrogen bond network. We demonstrate that heating up can destroy large, low-energy clusters into small, high-energy one because of increasing defects of topological structures.

One concern is a lack of mathematical rigor in defining water structures. The simplicial complex in complex network may provide a tool to propose unified mathematical definition of water clusters. Other concern is that the quantitative relationship between the dynamics of water clusters and water properties need to be explored further.

As a fundamental building unit of hydrogen bond networks, water cluster can detect structural and density fluctuations on different length scales in liquid water and also provide a new insight to explain the nucleation process of ice, liquid–liquid coexistence in supercooled water and hydrogen bond structures in the vicinity of a hydrophobic solute. Furthermore, it will be interesting in the future to discretize continuous liquid water as an assembly of large clusters, hierarchically forming a bridge between isolated molecules and bulk matter.

## Methods

### Molecular dynamics simulations

The 100-thousand molecular dynamics simulations are conducted by LAMMPS software package to explore the properties of water clusters. The water model is modeled using SPC/E rigid water model^53^, which is widely utilized to simulate macroscopic properties and microscopic structures of liquid water. The bond length and the bond angle of water are fixed with SHAKE algorithm. The cutoff of nonbonded interactions is 1.0 nm, and the long-range Coulomb interactions are computed using a particle–particle particle–mesh solver (PPPM). The system contains 181,914 water molecules in a box with periodic boundary conditions. The initial box size is 28.0 nm along the x-direction, 10.0 nm along the y-direction and 20.0 nm along the z-direction. The equations of motion are integrated using velocity-Verlet algorithm^68^ with a time step of 1.0 fs. A Nose–Hoover thermostat and barostat are used to control the temperature and pressure, respectively. The equilibration runs using an NPT ensemble are 2.5 ns. The temperatures of the system are kept constant at 278 K, 298 K and 318 K, and the pressure is 1.0 atm. The production runs output 500 configurations every 10.0 fs for structural analysis.

The density, diffusion coefficient, viscosity and structural properties of simulated water agree well with the results of experiments and other molecular dynamic simulations, and the simulation data are shown in Table 1 and Fig. 1 of the supplementary material.

### Extended hierarchical cluster methods

**Figure 8:**
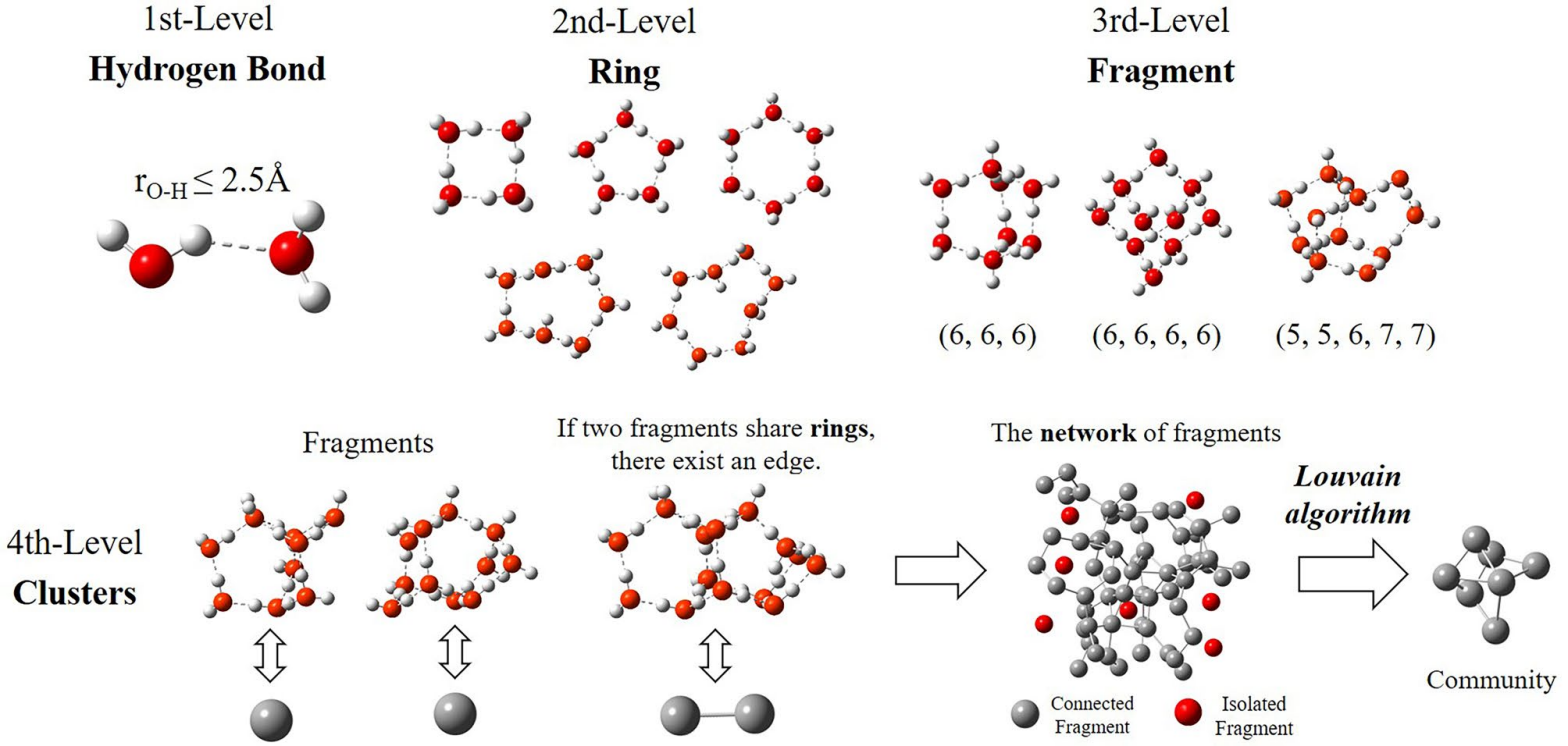
Schematics of the extended hierarchical clustering method. Hydrogen bonds, rings and fragments are considered 1st-, 2nd-, and 3rd-level structures, which are ball-and-stick models from a chemical perspective, and the red and white balls denote oxygen and hydrogen atoms, respectively. The full and dotted sticks denote O–H and hydrogen bonds, respectively. The clusters are illustrated from a topological perspective. The balls represent the graph community as water clusters. Note that the structures in the figure are only a selection among the considered structures by the clustering algorithm. The structures are drawn by GaussView 5.0.8^69^.

To detect specific topological structures in hydrogen-bonded networks, hydrogen bonds, rings and fragments, clusters are constructed as the first-, second-, third- and fourth-level structures of the water network using hierarchical cluster methods^47^, among which the clusters are an assembly of three-dimensional cage-like structures. To ensure that water clusters can largely cover hydrogen bond networks, we modify the definition of hierarchical cluster structures and propose extended hierarchical cluster method as illustrated in Fig. 8.

Hydrogen bonds, the first-level structures, are defined using a popular geometric standard in which two water molecules are regarded as hydrogen bonded when the distance between oxygen atoms of one molecule and the hydrogen atoms of another is less than 0.25 nm^70^. The rings, the second-level structures, are cyclic paths along hydrogen bonds investigated by “shortest-path” (SP) criterion^71^. The maximum ring size is limited to 8. The fragments are three-dimensional cage-like building blocks using topological definition proposed by Matsumoto^34^. The fragments combined by 3, 4, and 5 rings are taken into consideration. If two fragments share common rings, they have an edge, forming the network of fragments. The water clusters are identified as the best graph communities using Louvain algorithm^54,55^.

### Alpha shape of water clusters

The alpha shape is a formalization of the intuitive notion of shape for spatial point sets. An alpha shape is a concrete geometric model which is mathematically well-defined and unique for a given point set. The parameter alpha dictates the level of refinement, with a larger alpha resulting in coarser fits and a smaller alpha in finer fits. The volume and surface area of water clusters are based on the alpha shapes^56^ of water molecules in the clusters. The alpha shapes of clusters only take oxygen atoms into consideration. The alpha radius is chosen as 3.5 Å. The formation of alpha shape is implemented in MATLAB^72^.

## Supplementary Information

Supplementary Information.

## Data Availability

All data generated or analyzed during this study are included in this published article and its supplementary information files.
