# Supplementary material for: Water clusters and density fluctuations in liquid water based on extended hierarchical clustering methods
Source: Sci Rep. 2022 May 16;12:8036. doi: 10.1038/s41598-022-11947-6 (PMC9110331; doi:10.1038/s41598-022-11947-6)
Supplement: Supplementary file 1 — Supplementary Information. [file 41598_2022_11947_MOESM1_ESM.pdf]

Supplemental material for

"Water Clusters and Density Fluctuations in Liquid water Based on Extended Hierarchical Clustering Methods"

Yitian Gao, Hongwei Fang, Ke Ni, Yixuan Feng

State Key Laboratory of Hydro-science and Engineering, Department of Hydraulic Engineering, Tsinghua University, Beijing 100084, China

I. The Comparison of Experiments and Simulation Results.

The density, diffusion coefficient and viscosity of liquid water simulated by SPC/E at 278 K, 298 K and 318 K agree well with the results of experiments and other molecular dynamic simulations charted in **Table 1**.

Figure 1 shows the comparison of the radial distribution function (RDF) and angular distribution function (ADF) of liquid water at 278 K, 298 K and 318 K to assess the structural predictions of SPC/E. The RDF and ADF of SPC/E model is in good agreement with experiments[11,12] and other molecular dynamics simulations. The SPC/E respectively predicts the first, second, and third coordination shells of RDF at r = 2.8 Å, 4.5 Å and 6.8 Å corresponding to experimental RDF[12]. The first peaks of SPC/E are slightly higher than experiments to simulate the over-structuring of first shell in liquid water. All ADF shows a small shoulder at ~60° and a broad and strong peak at ~100°, whereas the ADF intensities of SPC/E models underestimate the small shoulder. The coordination numbers of SPC/E model are respectively 4.176, 4.153 and 4.110 at 278K, 298 K and 318 K within the range of 3.25 Å, similar to the experimental result, 4.62~4.67[11].

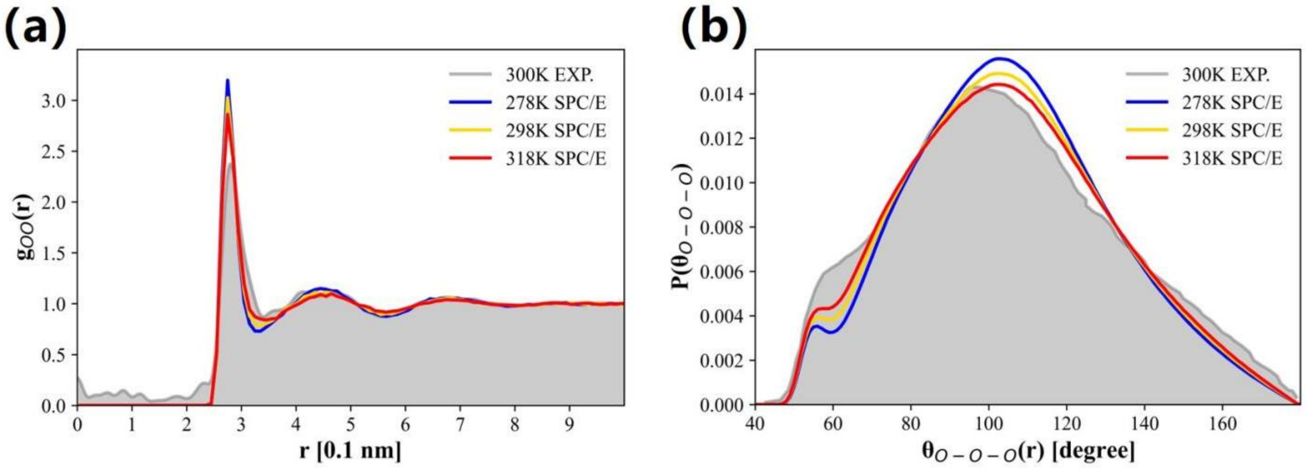

Figure 1. The comparison of radial and angular distance functions between experiments and SPC/E results at various temperatures.

Table 1. The comparison of physical properties between experiments and SPC/E results.

T(K)	Density (g/cm^3)			Diffusion coefficients (10^{-9} m^2/s)			Viscosity (mPa·s)		
	SPC/E	EXP.[1]	MD	SPC/E	EXP.[6]	MD	SPC/E	EXP.[10]	MD
**278**	1.0062	1.0000	1.0160 (282K SPC/E)[2]	1.853	1.313	1.510 (282K SPC/E)[2]	1.176	1.519	1.010 (283K SPC/E)[4]<br>1.450 (283K TIP5P)[4]
**298**	0.9983	0.9971	0.9984 (298K SPC/E)[3]<br>0.9937 (300K SPC/E)[4]<br>0.9781 (300K TIP5P)[4]	2.690	2.299	0.098 (300K CPMD)[7]<br>2.700 (300K AIMD)[7]<br>2.760 (298K SPC/E)[8]<br>5.650 (297K TIP3P)[8]	0.738	0.890	0.722 (300K SPC/E)[4]<br>0.708 (300K TIP5P)[4]
**318**	0.9876	0.9902	0.9851 (318K SPC/E)[5]<br>0.9920 (318K TIP4P)[5]	3.931	3.575	0.800 (316K BLYP)[9]	0.541	0.596	0.565 (313K TIP5P)[4]<br>0.476 (313K TIP5P)[4]

II. The Distribution and Lifetime of Hierarchical Water Structures.

First-level structure: hydrogen bond. According to SPC/E model, over 60.0% of water molecules are fully coordinated, which decrease with heating up. The other types of water molecules increase with temperature increasing. Increasing temperature accelerates the destruction of hydrogen bonds, as a result of which average lifetimes of hydrogen bonds obviously decrease from 11.79 ps (278 K), 7.99 ps (298 K) to 5.84 ps (318 K).

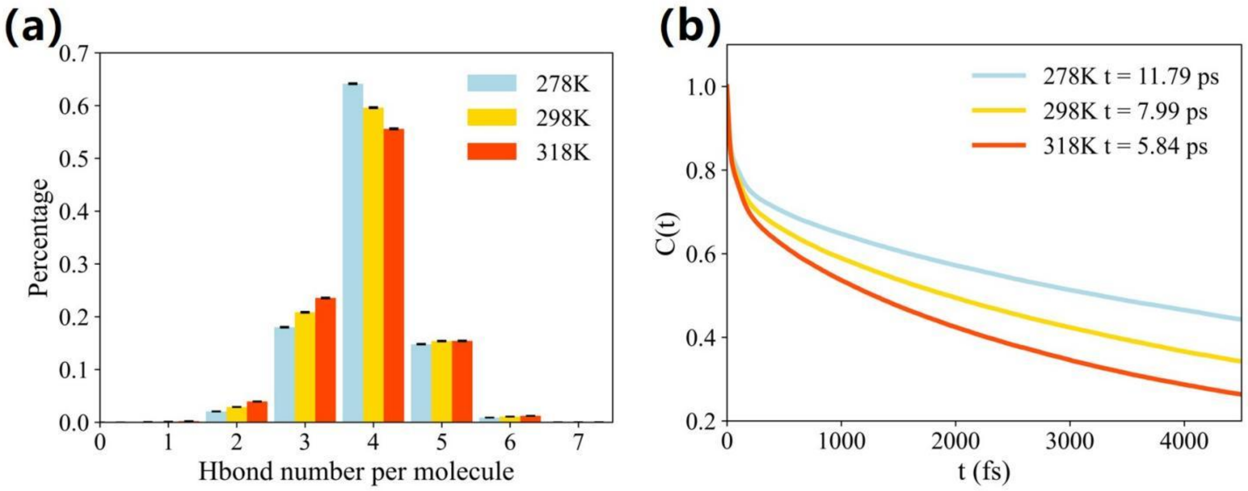

Figure 2. The distribution and lifetime of hydrogen bonds at various temperatures.

Second-level structure: ring. The 6-membered rings are mostly favored in all simulated cases, followed by 7-membered rings, 5-membered rings. With heating up, the number of rings decreases. The average lifetime of 5-membered rings is the longest rings, followed by 6-membered rings and 4-membered rings. Despite small quantity of 4-membered rings, the lifetimes of 4-membered rings is longer than 7- and 8-membered rings.

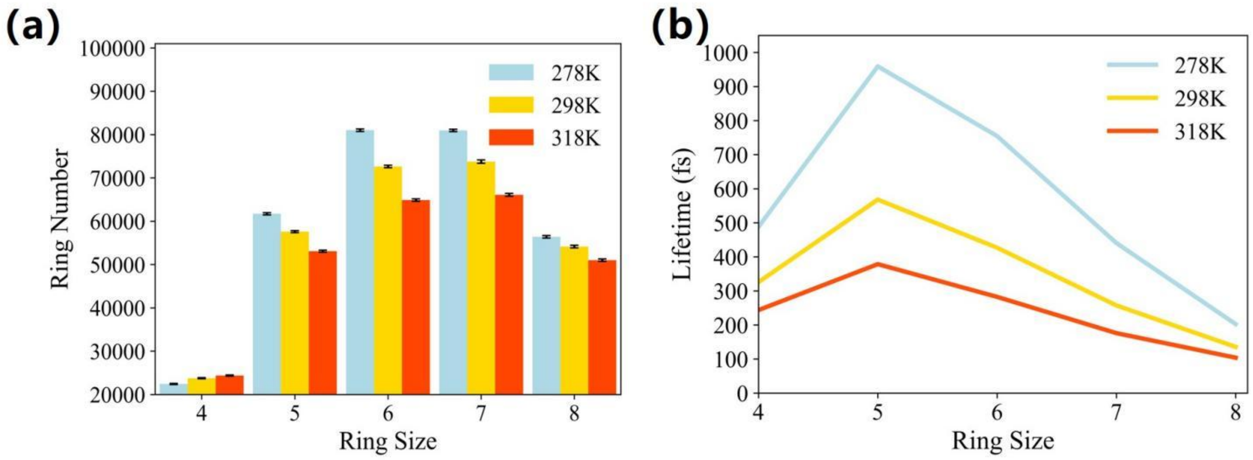

Figure 3. The distribution and lifetime of rings at various temperatures.

Third-level structure: fragment. Fragment is a compact assembly of several 4-, 5- ,6- , 7- and 8-membered rings. Entangled, distorted networks are interstitial between water clusters. Most of the

fragments have 9, 10 and 11 molecules. Several favored patterns of fragments involve: ① 3-ring fragments: (6,7,7), (6,8,8), (4,7,7), (4,6,6), (4,8,8); ② 4-ring fragments: (6,6,7,7), (5,6,6,7), (4,6,7,7), (5,5,7,7), (5,5,6,6), (4,5,6,7); ③ 5-ring fragments: (5,6,6,7,8), (4,5,6,6,7), (4,6,6,7,7), (5,6,6,6,7), (5,6,6,7,8), (6,6,6,7,7). The lifetimes of fragments are related with the fragment numbers. Instead, several topological structures of fragments correspond to energetic stability. For instance, the average lifetime of the fragments (6,6,6) is 190.52 fs, followed by (6,7,7) and (4,6,6). The long-lifetime 4-ring fragments are (5,6,6,7), (6,6,6,8) and (4,5,6,7). And the long-lifetime 5-ring fragments are (5,5,5,5,6), (6,6,6,6,6) and (5,6,6,6,7).

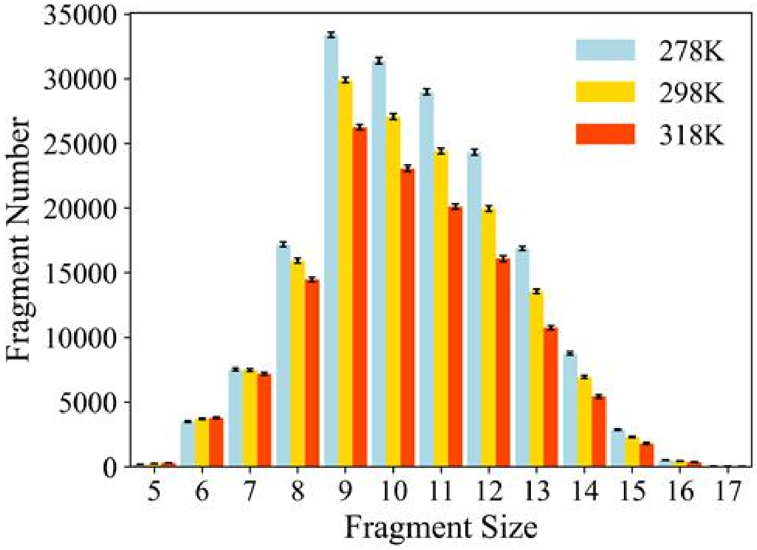

Figure 4. The distribution of fragments at various temperatures.

Table 2. The distribution of fragments at various temperatures.

Type	278 K	298 K	318 K	Lifetime(fs 298K)
**(4, 4, 4)**	196.99	250.99	305.28	45.43
**(4, 5, 5)**	3475.31	3689.85	3768.90	86.71
**(4, 6, 6)**	7058.95	6920.12	6571.45	103.68
**(4, 7, 7)**	8907.78	8744.62	8267.46	89.52
**(4, 8, 8)**	7241.14	7481.43	7409.74	68.59
**(6, 6, 6)**	5973.69	4887.53	4017.92	190.52
**(6, 7, 7)**	16857.95	13992.03	11450.12	148.66
**(6, 8, 8)**	10677.56	9424.18	8274.29	89.55
**(8, 8, 8)**	1609.92	1551.17	1418.35	68.85
**(4, 4, 4, 4)**	11.73	15.36	20.40	27.56
**(4, 4, 4, 6)**	129.35	155.60	164.23	89.81
**(4, 4, 5, 5)**	345.55	404.67	449.61	42.21
**(4, 4, 6, 6)**	754.09	801.72	807.30	58.78
**(4, 4, 7, 7)**	971.43	1045.70	1036.39	54.11
**(4, 4, 8, 8)**	825.67	919.82	967.65	46.64

**(4, 5, 5, 6)**	1108.83	1051.85	978.27	79.68
**(4, 5, 6, 7)**	3135.38	2882.78	2525.97	96.15
**(4, 6, 6, 6)**	1462.14	1300.72	1125.04	82.49
**(4, 6, 6, 8)**	1688.41	1497.48	1322.20	91.56
**(4, 6, 7, 7)**	4442.16	3966.77	3420.46	75.06
**(4, 6, 8, 8)**	3029.27	2857.55	2591.45	60.06
**(4, 7, 7, 8)**	957.71	901.91	796.84	63.06
**(4, 8, 8, 8)**	554.93	558.25	522.47	49.40
**(5, 5, 5, 5)**	427.70	417.37	382.55	64.63
**(5, 5, 6, 6)**	3406.57	2869.43	2386.15	91.35
**(5, 5, 7, 7)**	3418.72	2905.90	2430.95	77.10
**(5, 5, 8, 8)**	2243.94	2045.37	1818.20	58.29
**(5, 6, 6, 7)**	6475.45	5017.52	3849.04	119.82
**(5, 6, 7, 8)**	3707.88	3007.79	2378.02	89.90
**(5, 7, 7, 7)**	1475.74	1192.28	929.21	92.11
**(5, 7, 8, 8)**	2223.67	1932.69	1621.48	64.38
**(6, 6, 6, 6)**	1878.52	1505.03	1195.09	87.49
**(6, 6, 6, 8)**	1827.82	1411.74	1092.75	96.44
**(6, 6, 7, 7)**	7720.47	6018.89	4635.02	85.15
**(6, 6, 8, 8)**	4790.77	3943.21	3268.02	62.85
**(6, 7, 7, 8)**	2844.91	2307.20	1828.16	76.83
**(6, 8, 8, 8)**	945.55	832.08	712.83	52.57
**(7, 7, 7, 7)**	1880.87	1547.22	1230.90	65.21
**(7, 7, 8, 8)**	2845.37	2505.42	2138.13	52.07
**(8, 8, 8, 8)**	622.80	597.72	550.33	41.73
**(4, 4, 4, 4, 6)**	5.44	5.21	6.55	51.21
**(4, 4, 4, 5, 5)**	14.52	17.09	19.96	28.51
**(4, 4, 4, 5, 7)**	39.62	43.01	44.59	69.79
**(4, 4, 4, 6, 6)**	31.09	35.63	36.90	50.03
**(4, 4, 4, 6, 8)**	49.45	51.45	53.95	60.90
**(4, 4, 4, 7, 7)**	46.37	53.49	54.66	47.04
**(4, 4, 4, 8, 8)**	46.82	55.87	62.15	35.93
**(4, 4, 5, 5, 6)**	201.64	196.13	194.37	58.54
**(4, 4, 5, 6, 7)**	522.27	521.15	475.09	61.93
**(4, 4, 5, 7, 8)**	152.98	160.76	147.63	59.09
**(4, 4, 6, 6, 6)**	217.58	206.92	184.21	67.24
**(4, 4, 6, 6, 8)**	267.35	261.55	242.89	61.72
**(4, 4, 6, 7, 7)**	616.33	594.61	528.81	53.82
**(4, 4, 6, 8, 8)**	469.24	467.76	439.65	44.39
**(4, 4, 7, 7, 8)**	166.58	166.21	159.33	44.84
**(4, 4, 8, 8, 8)**	75.96	80.72	81.35	39.47
**(4, 5, 5, 5, 5)**	55.16	55.26	48.97	49.88
**(4, 5, 5, 5, 7)**	245.95	215.46	180.94	74.09

**(4, 5, 5, 6, 6)**	752.68	655.28	562.99	64.91
**(4, 5, 5, 6, 8)**	246.98	220.65	188.01	69.06
**(4, 5, 5, 7, 7)**	745.88	656.90	566.59	55.89
**(4, 5, 5, 8, 8)**	511.73	486.78	440.49	47.45
**(4, 5, 6, 6, 7)**	2393.51	2001.53	1614.59	68.50
**(4, 5, 6, 7, 8)**	1617.08	1412.72	1185.77	57.19
**(4, 5, 7, 7, 7)**	837.63	711.72	569.95	57.93
**(4, 5, 7, 8, 8)**	986.49	904.49	792.37	46.84
**(4, 6, 6, 6, 6)**	352.91	291.33	234.72	70.30
**(4, 6, 6, 6, 8)**	985.17	787.93	649.37	65.78
**(4, 6, 6, 7, 7)**	2037.20	1669.47	1314.14	59.04
**(4, 6, 6, 8, 8)**	1215.40	1039.40	889.45	49.53
**(4, 6, 7, 7, 8)**	1742.22	1490.91	1213.45	51.91
**(4, 6, 8, 8, 8)**	531.27	482.08	421.60	40.67
**(4, 7, 7, 7, 7)**	418.16	346.02	281.67	51.57
**(4, 7, 7, 8, 8)**	769.30	705.27	605.76	41.18
**(4, 8, 8, 8, 8)**	151.08	149.48	140.83	33.80
**(5, 5, 5, 5, 6)**	151.35	123.59	93.93	92.30
**(5, 5, 5, 6, 7)**	862.72	653.73	489.10	81.42
**(5, 5, 5, 7, 8)**	373.77	299.58	234.13	69.37
**(5, 5, 6, 6, 6)**	728.04	525.23	374.66	80.49
**(5, 5, 6, 6, 8)**	653.65	499.71	372.08	71.18
**(5, 5, 6, 7, 7)**	1987.76	1491.02	1084.49	71.03
**(5, 5, 6, 8, 8)**	1114.33	902.65	708.04	54.40
**(5, 5, 7, 7, 8)**	693.92	550.31	419.32	58.60
**(5, 5, 8, 8, 8)**	179.20	159.73	132.70	43.06
**(5, 6, 6, 6, 7)**	2111.13	1480.62	1032.10	83.74
**(5, 6, 6, 7, 8)**	2348.67	1748.72	1290.22	62.24
**(5, 6, 7, 7, 7)**	1888.57	1357.88	966.48	66.63
**(5, 6, 7, 8, 8)**	1970.59	1571.32	1215.58	49.05
**(5, 7, 7, 7, 8)**	701.40	533.77	401.99	51.66
**(5, 7, 8, 8, 8)**	375.70	315.42	257.74	43.46
**(6, 6, 6, 6, 6)**	272.36	193.84	132.36	88.91
**(6, 6, 6, 6, 8)**	572.07	395.11	288.85	62.99
**(6, 6, 6, 7, 7)**	2044.53	1408.06	965.92	68.86
**(6, 6, 6, 8, 8)**	1136.32	830.77	627.92	51.09
**(6, 6, 7, 7, 8)**	1775.25	1298.77	949.96	54.80
**(6, 6, 8, 8, 8)**	506.23	407.89	322.93	42.61
**(6, 7, 7, 7, 7)**	221.55	170.33	128.07	55.80
**(6, 7, 7, 8, 8)**	213.12	188.65	155.66	44.96
**(6, 8, 8, 8, 8)**	37.37	35.54	32.35	39.12
**(7, 7, 7, 7, 8)**	221.55	170.33	128.07	45.84
**(7, 7, 8, 8, 8)**	213.12	188.65	155.66	38.69

(8, 8, 8, 8, 8)	37.37	35.54	32.35	34.68

III. The Topology of Water Clusters.

The connection patterns of fragments in 2- to 6-fragment clusters are charted below.

Table 3. The topological patterns of 2-fragment clusters at various temperatures.

Rank	278K			298K			318K		
	Fragment 1	Fragment 2	Number	Fragment 1	Fragment 2	Number	Fragment 1	Fragment 2	Number
**1**	(4,7,7)	(4,8,8)	21.776	(4,7,7)	(4,8,8)	30.906	(4,7,7)	(4,8,8)	38.154
**2**	(6,7,7)	(6,8,8)	11.488	(4,8,8)	(4,8,8)	16.582	(4,8,8)	(4,8,8)	22.45
**3**	(4,8,8)	(4,8,8)	11.094	(4,6,6)	(4,8,8)	13.28	(4,6,6)	(4,8,8)	16.636
**4**	(4,6,6)	(4,8,8)	9.546	(6,7,7)	(6,8,8)	13.016	(4,7,7)	(4,7,7)	16.096
**5**	(6,7,7)	(6,7,7)	9.41	(4,7,7)	(4,7,7)	12.404	(4,6,6)	(4,7,7)	15.85
**6**	(4,6,6)	(4,7,7)	8.85	(4,6,6)	(4,7,7)	12.25	(6,7,7)	(6,8,8)	14.36
**7**	(4,7,7)	(4,7,7)	8.616	(6,7,7)	(6,7,7)	11.378	(6,7,7)	(6,7,7)	12.56
**8**	(5,5,6,6)	(5,6,6,7)	8.534	(6,7,7)	(5,6,6,7)	9.748	(4,5,5)	(4,8,8)	11.98
**9**	(6,7,7)	(5,6,6,7)	8.254	(6,8,8)	(6,8,8)	9.602	(6,8,8)	(6,8,8)	11.066
**10**	(6,8,8)	(6,8,8)	6.892	(4,7,7)	(4,5,6,7)	9.034	(4,7,7)	(4,5,6,7)	10.74

Table 4. The topological patterns of 3-fragment clusters at various temperatures.

Temperature (K)	Rank	Topological patterns			
		Fragment 1	Fragment 2	Fragment 3	Number
**278**	1	(4,7,7)	(4,8,8)	(4,8,8)	0.744
	2	(5,5,6,6)	(5,6,6,7)	(5,6,6,7)	0.478
	3	(4,6,7,7)	(4,6,7,7)	(6,6,7,7)	0.448
	4	(4,7,7)	(4,7,7)	(4,8,8)	0.43
	5	(5,5,6,6)	(5,5,6,6)	(5,6,6,7)	0.426
	6	(4,6,7,7)	(6,6,7,7)	(4,5,6,6,7)	0.424
	7	(5,6,7,8)	(5,6,7,8)	(6,6,8,8)	0.372
	8	(4,7,7)	(4,8,8)	(4,5,6,7)	0.36
	9	(6,7,7)	(5,5,6,6)	(5,6,6,7)	0.352
	10	(5,5,6,6)	(5,5,7,7)	(5,6,6,7)	0.35
**298**	1	(4,7,7)	(4,8,8)	(4,8,8)	0.9
	2	(4,7,7)	(4,7,7)	(4,8,8)	0.676
	3	(4,6,7,7)	(6,6,7,7)	(4,5,6,6,7)	0.642
	4	(4,6,7,7)	(4,6,7,7)	(6,6,7,7)	0.5
	5	(4,6,6)	(4,7,7)	(4,8,8)	0.5
	6	(5,5,6,6)	(5,6,6,7)	(5,6,6,7)	0.478
	7	(6,7,7)	(5,5,6,6)	(5,6,6,7)	0.476
	8	(4,7,7)	(4,8,8)	(4,5,6,7)	0.42

	9	(5,5,6,6)	(5,5,6,6)	(5,6,6,7)	0.414
	10	(4,6,7,7)	(6,6,7,7)	(6,6,7,7)	0.414
**318**	1	(4,7,7)	(4,8,8)	(4,8,8)	1.346
	2	(4,7,7)	(4,7,7)	(4,8,8)	0.876
	3	(4,6,7,7)	(6,6,7,7)	(4,5,6,6,7)	0.748
	4	(4,6,7,7)	(4,6,7,7)	(6,6,7,7)	0.622
	5	(4,8,8)	(4,8,8)	(4,8,8)	0.612
	6	(4,6,7,7)	(6,6,7,7)	(6,6,7,7)	0.544
	7	(4,6,6)	(4,7,7)	(4,8,8)	0.536
	8	(4,7,7)	(4,8,8)	(4,5,6,7)	0.522
	9	(5,5,6,6)	(5,6,6,7)	(5,6,6,7)	0.494
	10	(5,5,6,6)	(5,5,6,6)	(5,6,6,7)	0.466

Table 5. The topological patterns of 4-fragment clusters at various temperatures.

Temperature (K)	Rank	Topological patterns				
		Fragment 1	Fragment 2	Fragment 3	Fragment 4	Number
**278**	1	(4,6,6)	(4,7,7)	(6,7,7)	(6,7,7)	1.558
	2	(4,7,7)	(4,7,7)	(6,7,7)	(6,7,7)	1.414
	3	(4,8,8)	(4,8,8)	(6,8,8)	(6,8,8)	1.32
	4	(4,6,6)	(4,8,8)	(6,8,8)	(6,8,8)	1.248
	5	(6,7,7)	(6,7,7)	(6,7,7)	(6,7,7)	1.204
	6	(4,6,6)	(4,6,6)	(6,6,6)	(6,6,6)	0.624
	7	(6,8,8)	(6,8,8)	(6,8,8)	(6,8,8)	0.622
	8	(4,8,8)	(4,8,8)	(8,8,8)	(8,8,8)	0.586
	9	(6,7,7)	(6,7,7)	(5,5,7,7)	(5,5,7,7)	0.472
	10	(6,8,8)	(6,8,8)	(8,8,8)	(8,8,8)	0.466
**298**	1	(4,6,6)	(4,7,7)	(6,7,7)	(6,7,7)	2.302
	2	(4,8,8)	(4,8,8)	(6,8,8)	(6,8,8)	2.296
	3	(4,7,7)	(4,7,7)	(6,7,7)	(6,7,7)	1.902
	4	(4,6,6)	(4,8,8)	(6,8,8)	(6,8,8)	1.796
	5	(6,7,7)	(6,7,7)	(6,7,7)	(6,7,7)	1.168
	6	(4,6,6)	(4,6,6)	(6,6,6)	(6,6,6)	0.872
	7	(4,8,8)	(4,8,8)	(8,8,8)	(8,8,8)	0.834
	8	(4,7,7)	(4,7,7)	(4,7,7)	(4,7,7)	0.802
	9	(4,8,8)	(4,8,8)	(4,8,8)	(4,8,8)	0.764
	10	(6,8,8)	(6,8,8)	(6,8,8)	(6,8,8)	0.712
**318**	1	(4,6,6)	(4,7,7)	(6,7,7)	(6,7,7)	3.556
	2	(4,6,6)	(4,8,8)	(6,8,8)	(6,8,8)	2.962
	3	(4,8,8)	(4,8,8)	(6,8,8)	(6,8,8)	2.894
	4	(4,7,7)	(4,7,7)	(6,7,7)	(6,7,7)	2.674
	5	(6,7,7)	(6,7,7)	(6,7,7)	(6,7,7)	1.364
	6	(4,8,8)	(4,8,8)	(4,8,8)	(4,8,8)	1.224

	7	(4,8,8)	(4,8,8)	(8,8,8)	(8,8,8)	1.182
	8	(4,6,6)	(4,6,6)	(6,6,6)	(6,6,6)	1.13
	9	(6,8,8)	(6,8,8)	(6,8,8)	(6,8,8)	1.018
	10	(4,7,7)	(4,7,7)	(4,7,7)	(4,7,7)	0.99

Table 6. The topological patterns of 5-fragment clusters at various temperatures.

Temperature (K)	Rank	Topological patterns					
		Fragment 1	Fragment 2	Fragment 3	Fragment 4	Fragment 5	Number
**278**	1	(4,5,6,7)	(4,5,6,7)	(4,6,7,7)	(5,6,6,7)	(5,6,6,7)	0.156
	2	(5,6,7,8)	(5,6,7,8)	(6,6,6,8)	(6,6,6,8)	(6,6,8,8)	0.126
	3	(4,8,8)	(4,8,8)	(6,8,8)	(6,8,8)	(6,8,8)	0.092
	4	(4,6,6)	(4,7,7)	(4,7,7)	(6,7,7)	(6,7,7)	0.078
	5	(4,6,6)	(4,7,7)	(4,8,8)	(6,7,7)	(6,7,7)	0.078
	6	(4,6,6)	(4,8,8)	(4,8,8)	(6,8,8)	(6,8,8)	0.074
	7	(4,8,8)	(4,8,8)	(4,8,8)	(6,8,8)	(6,8,8)	0.07
	8	(4,7,7)	(4,7,7)	(4,8,8)	(6,7,7)	(6,7,7)	0.06
	9	(4,7,7)	(4,7,7)	(4,7,7)	(4,7,7)	(4,8,8)	0.058
	10	(4,7,7)	(4,7,7)	(6,7,7)	(6,7,7)	(4,5,6,7)	0.058
**298**	1	(4,5,6,7)	(4,5,6,7)	(4,6,7,7)	(5,6,6,7)	(5,6,6,7)	0.216
	2	(4,7,7)	(4,8,8)	(4,8,8)	(6,8,8)	(6,8,8)	0.16
	3	(4,8,8)	(4,8,8)	(4,8,8)	(6,8,8)	(6,8,8)	0.146
	4	(4,8,8)	(4,8,8)	(4,8,8)	(4,8,8)	(4,8,8)	0.142
	5	(4,6,6)	(4,8,8)	(4,8,8)	(6,8,8)	(6,8,8)	0.124
	6	(4,7,7)	(4,8,8)	(4,8,8)	(4,8,8)	(4,8,8)	0.116
	7	(4,7,7)	(4,7,7)	(4,7,7)	(4,7,7)	(4,8,8)	0.104
	8	(5,6,7,8)	(5,6,7,8)	(6,6,6,8)	(6,6,6,8)	(6,6,8,8)	0.102
	9	(5,6,7,8)	(5,6,7,8)	(5,6,7,8)	(5,6,7,8)	(6,6,8,8)	0.098
	10	(4,7,7)	(4,7,7)	(4,7,7)	(6,7,7)	(6,7,7)	0.09
**318**	1	(4,5,6,7)	(4,5,6,7)	(4,6,7,7)	(5,6,6,7)	(5,6,6,7)	0.272
	2	(4,8,8)	(4,8,8)	(4,8,8)	(6,8,8)	(6,8,8)	0.22
	3	(4,7,7)	(4,8,8)	(4,8,8)	(4,8,8)	(4,8,8)	0.198
	4	(4,7,7)	(4,8,8)	(4,8,8)	(6,8,8)	(6,8,8)	0.19
	5	(4,8,8)	(4,8,8)	(4,8,8)	(4,8,8)	(4,8,8)	0.186
	6	(4,6,6)	(4,8,8)	(4,8,8)	(6,8,8)	(6,8,8)	0.168
	7	(4,7,7)	(4,7,7)	(4,7,7)	(4,7,7)	(4,8,8)	0.166
	8	(4,6,6)	(4,7,7)	(4,7,7)	(6,7,7)	(6,7,7)	0.138
	9	(4,7,7)	(4,7,7)	(4,7,7)	(4,7,7)	(4,7,7)	0.138
	10	(4,7,7)	(4,7,7)	(4,7,7)	(6,7,7)	(6,7,7)	0.13

Table 7. The topological patterns of 6-fragment clusters at various temperatures.

Temperature (K)	Rank	Topological patterns						
		Fragment 1	Fragment 2	Fragment 3	Fragment 4	Fragment 5	Fragment 6	Number
278	1	(4,5,5,6)	(4,7,7,8)	(5,6,6,7)	(4,4,6,8,8)	(4,6,6,6,8)	(4,6,6,6,8)	0.028
	2	(5,5,6,6)	(5,5,6,6)	(5,5,6,6)	(5,5,6,6)	(4,4,6,8,8)	(4,4,6,8,8)	0.026
	3	(4,6,8,8)	(5,5,6,6)	(5,5,8,8)	(8,8,8,8)	(4,6,6,8,8)	(6,6,6,8,8)	0.026
	4	(6,7,7)	(6,7,7)	(4,5,6,7)	(4,5,6,7)	(5,5,7,7)	(5,6,6,7)	0.024
	5	(6,7,7)	(4,6,7,7)	(5,5,7,7)	(5,6,7,8)	(4,5,5,6,6)	(4,5,6,6,7)	0.022
	6	(4,6,8,8)	(6,6,6,8)	(6,6,6,8)	(6,6,6,8)	(6,6,8,8)	(6,6,8,8)	0.022
	7	(4,5,6,7)	(4,6,8,8)	(4,6,6,7,7)	(4,6,6,8,8)	(6,6,6,7,7)	(6,6,6,8,8)	0.022
	8	(5,6,6,7)	(5,6,7,8)	(5,6,7,8)	(6,6,6,8)	(6,6,8,8)	(6,6,8,8)	0.022
	9	(5,5,6,6)	(5,5,8,8)	(5,5,8,8)	(4,6,6,8,8)	(4,6,6,8,8)	(4,6,6,8,8)	0.02
	10	(4,8,8)	(6,7,7)	(4,6,6,6)	(5,5,6,6)	(5,5,5,5,6)	(5,6,6,7,8)	0.02
298	1	(4,4,6,6)	(4,6,7,7)	(4,6,7,7)	(5,5,6,6)	(5,5,7,7)	(6,6,7,7)	0.022
	2	(4,7,7)	(4,6,7,7)	(4,6,7,7)	(4,6,7,7)	(5,5,7,7)	(5,5,7,7)	0.022
	3	(4,6,6)	(4,7,7)	(4,8,8)	(4,8,8)	(4,8,8)	(4,8,8)	0.022
	4	(5,6,6,7)	(5,6,7,8)	(5,6,7,8)	(6,6,6,8)	(6,6,8,8)	(6,6,8,8)	0.022
	5	(5,6,7,8)	(5,6,7,8)	(6,6,6,8)	(6,6,6,8)	(6,6,7,7)	(6,6,8,8)	0.022
	6	(5,5,5,5)	(5,5,6,6)	(5,5,6,6)	(5,5,7,7)	(5,6,6,7)	(6,6,6,6)	0.022
	7	(6,7,7)	(4,5,6,7)	(4,5,6,7)	(4,6,7,7)	(5,6,6,7)	(5,6,6,7)	0.02
	8	(4,6,6)	(4,8,8)	(4,4,4,6)	(4,5,6,7)	(5,5,6,6)	(5,5,6,6,8)	0.02
	9	(4,6,6,6)	(4,6,8,8)	(6,6,8,8)	(4,6,6,6,8)	(4,6,6,6,8)	(6,6,6,6,8)	0.018
	10	(4,7,7)	(4,8,8)	(4,8,8)	(4,8,8)	(4,8,8)	(4,8,8)	0.018
318	1	(4,6,6,6)	(4,6,8,8)	(4,6,8,8)	(4,6,8,8)	(6,6,8,8)	(6,6,8,8)	0.034
	2	(4,5,6,7)	(4,5,6,7)	(4,6,7,7)	(5,6,6,7)	(5,6,6,7)	(5,6,6,7)	0.028
	3	(4,5,6,7)	(4,5,6,7)	(4,5,6,7)	(4,6,7,7)	(5,6,6,7)	(5,6,6,7)	0.026
	4	(4,7,7)	(4,8,8)	(4,8,8)	(4,8,8)	(4,8,8)	(4,8,8)	0.022
	5	(4,5,6,7)	(4,4,6,7,7)	(4,6,6,7,7)	(6,7,7,7,7)	(6,7,7,8,8)	(6,7,7,8,8)	0.022
	6	(6,7,7)	(4,4,5,5)	(4,6,7,7)	(5,5,7,7)	(5,5,7,7)	(6,6,7,7)	0.022
	7	(4,8,8)	(4,8,8)	(4,8,8)	(4,8,8)	(4,6,7,7)	(5,6,6,7)	0.02
	8	(4,5,6,7)	(5,6,7,8)	(5,6,7,8)	(5,6,7,8)	(5,6,7,8)	(6,6,8,8)	0.02
	9	(6,7,7)	(6,7,7)	(6,6,7,7)	(6,6,8,8)	(7,7,7,7)	(7,7,8,8)	0.018
	10	(4,8,8)	(4,4,7,7)	(4,4,8,8)	(6,7,7,8)	(7,7,8,8)	(4,6,7,7,8)	0.018

IV. The Isosbestic Point of Water Clusters Distributions.

As shown in Fig.5, the distributions of $S > 100$ form a shoulder at low temperature. Besides, S_c denotes an isosbestic point dividing the size of clusters with opposite temperature responses. With heating up, the cluster numbers decrease when $S > S_c$ but increase when $S < S_c$, indicating that large clusters are destroyed into small ones due to stronger thermal fluctuation caused by higher temperatures.

The cluster distributions are influenced by simulated box size dimensions. The other boxes (respectively named as box II and III), the length of which is 0.75 and 0.5 times as big as the present box (named as box I), are simulated in the same processes. The isobestic points are respectively located at S_c = 100~120, 180~200 and 230~270 in the box III, II and I, which are positively proportional to the box size, $S_c \propto L$. The limitation of periodic boundaries can deviate the number of large clusters when $S > 100$. In a big enough box, maximum clusters may probably form percolating networks which causes so-called isobestic points vanishing.

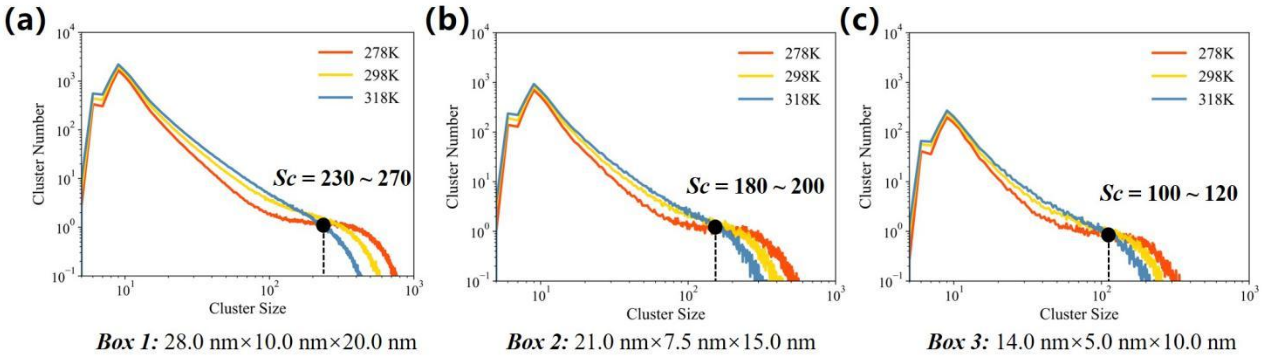

Figure 5. The distribution of water clusters at various temperatures in different boxes.

REFERENCES

1. Kell, G. S. Precise Representation of Volume Properties of Water at One Atmosphere. J. Chem. Eng. Data 12, 66 – 69 (1967).
2. Báez, L. A. & Clancy, P. Existence of a density maximum in extended simple point charge water. J. Chem. Phys. 101, 9837 – 9840 (1994).
3. Bryk, T. & Haymet, A. D. J. The ice/water interface: Density-temperature phase diagram for the SPC/E model of liquid water. Mol. Simul. 30, 131 – 135 (2004).
4. Song, Y. & Dai, L. L. The shear viscosities of common water models by non-equilibrium molecular dynamics simulations. 7022, (2010).
5. Bedrov, D. & Smith, G. D. Thermal conductivity of molecular fluids from molecular dynamics simulations: Application of a new imposed-flux method. J. Chem. Phys. 113, 8080 – 8084 (2000).
6. Holz, M., Heil, S. R. & Sacco, A. Temperature-dependent self-diffusion coefficients of water and six selected molecular liquids for calibration in accurate 1H NMR PFG measurements. Phys. Chem. Chem. Phys. 2, 4740 – 4742 (2000).
7. Liu, J., He, X. & Zhang, J. Z. H. Structure of liquid water-a dynamical mixture of tetrahedral and ‘ring-and-chain’ like structures. Phys. Chem. Chem. Phys. 19, 11931 – 11936 (2017).
8. Mark, P. & Nilsson, L. Structure and dynamics of the TIP3P, SPC, and SPC/E water models at 298 K. J. Phys. Chem. A 105, 9954 – 9960 (2001).
9. Lin, I. C., Seitsonen, A. P., Coutinho-Neto, M. D., Tavernelli, I. & Rothlisberger, U. Importance of van der Waals interactions in liquid water. J. Phys. Chem. B 113, 1127 – 1131 (2009).
10. Kestin, J., Sokolov, M. & Wakeham, W. A. Viscosity of liquid water in the range −8 °C to 150 °C. J. Phys. Chem. Ref. Data 7, 941 – 948 (1978).

11. Skinner, L. B. et al. Benchmark oxygen-oxygen pair-distribution function of ambient water from x-ray diffraction measurements with a wide Q-range. J. Chem. Phys. 138, (2013).
12. Soper, A. K. & Benmore, C. J. Quantum differences between heavy and light water. Phys. Rev. Lett. 101, 1 – 4 (2008).
